# Effect of trans-retinoic acid in the inhibition of cholesteatoma in guinea pigs

**DOI:** 10.1016/S1808-8694(15)30751-5

**Published:** 2015-10-19

**Authors:** Marcos Luiz Antunes, Yotaka Fukuda, Norma de Oliveira Penido, Rimarcs Ferreira

**Affiliations:** 1 Doctoral thesis, UNIFESP; Professor at the ABC Medical School; 2 Habilitation degree (livre docência) Professor, UNIFESP; Adjunct Professor, de Otorhinolaryngology and Head & Neck Surgery Department, UNIFESP; 3 Doctoral thesis, UNIFESP; Affilitated Professor Otorhinolaryngology and Head & Neck Surgery Department, UNIFESP; 4 Habilitation degree (livre docência) Professor, UNIFESP; Adjunct Professor, Pathology Department, UNIFESP

**Keywords:** guinea pig, middle ear cholesteatoma, prevention and control, chemically induced, chemoterapy

## Abstract

Middle ear cholesteatoma affected more than 5 million people until the 80's. Many animal models were used, unsuccessfully, to study an alternative therapy to cholesteatoma.

**Aim:**

observe the effect of the trans-retinoic acid in the inhibition of middle ear cholesteatomas induced by propylene glycol.

**Study Design:**

Clinical and Experimental.

**Methods:**

25 guinea pigs were submitted to the application of a 100% propylene glycol solution in their bulla bilaterally and a solution of trans-retinoic acid was applied locally in the external right ear, while in the left ear saline solution was applied (control ear). The guinea pigs were slaughtered and their temporal bones were prepared for macroscopic and histological analysis.

**Results:**

The macroscopic findings had evidenced the presence of cholesteatoma in 25% of the right ears and 85% of the left ears (P=0.0003 *). The histological study had evidenced the presence of cholesteatoma in 30% of right ears and 75% of the left ears (P=0.0104*).

**Conclusion:**

The local use of the trans-retinoic acid is effective in inhibiting the induced formation of cholesteatomas in guinea pigs.

## INTRODUCTION

Cholesteatomatous chronic otitis media affected an estimated over 5 million people worldwide in the 1980s.^1^ Cholesteatomas can migrate into and invade adjacent structures, resulting in bone erosion and destruction of the middle ear ossicles. Furthermore, the disease may relapse, and some patients require repeated surgery. Until the present there is no effective method to avoid cholesteatoma proliferation, invasion or recurrence.

Placing a mixture of talcum powder and fibrin in the bulla of guinea-pigs results in a typical cholesteatoma that originates from epidermal basal cells of the tympanic membrane and migrates into the middle ear, which corroborates the epithelial migration theory.^2^ Propylene glycol (PG) has been shown to cause epithelial migration and cholesteatomatous chronic otitis media in North-American chinchillas, in which optic drops (Cortisporin) containing PG (10%) was used.^3^ Increasing the dose of PG in topical preparations injected transtympanically yields cholesteatomas^4^^,^^5^ in a progressively higher percentage of rats, reaching 100% at a concentration of 90%. After producing a cholesteatoma in chinchillas by using PG (60%),^5^-fluorouracil was used to inhibit growth of the cholesteatoma, with satisfactory results.^6^ Hyaluronic acid was applied to the external ear of chinchillas in an attempt to inhibit PG-induced cholesteatoma development, with poor results.^7^ Similarly poor results were seen when using cyclophosphamide systemically in chinchillas.^8^

Retinoic acid appears to affect epidermal keratinization and secretory gland activity. Isotretinoin, a synthetic retinol analogue, was used systemically with poor cholesteatoma-inhibiting results.^9^ All-trans retinoic acid is used topically to treat acne vulgaris. It decreases epithelial cell adhesion and is effective in skin keratinization disorders; it also helps repair skin following exposure to phototherapy or other external agents. All-trans retinoic acid inhibited in vitro cholesteatoma migration in surgically removed human cholesteatomas kept in a growth-promoting culture.^10^

Considering that the cholesteatoma is a disease with multiple factors, that there are many theories about its etiology and pathogenesis, and that there is as yet no effective treatment for preventing epithelial migration and cholesteatoma formation in the middle ear, additional experimental studies are required to clarify these issues. The aim of this paper was to investigate the effects of all-trans retinoic acid used topically in guinea-pig external ears and the area adjacent to the tympanic membrane in inhibiting middle ear cholesteatoma formation by PG 100%.

## MATERIAL AND METHOD

Twenty-five male and female guinea pigs weighing between 400 and 600 grams, aged between 4 and 6 months, with no infection or history of disease, were used. The animals were provided by the Experimental Model Development Center for Medicine and Biology (Centro de Desenvolvimento de Modelos Experimentais para Medicina e Biologia). Animal rights laws defining the guidelines scientific experimentation and animal vivisections were followed; the Research Ethics Committee of the institution approved the study (process number 1199/02).

The animals were anesthetized with a ketamine chloridrate (70mg/kg) and xylazine chloridrate (6mg/kg) solution for intraperitoneal use. The external acoustic meatus or auditory canal was inspected microscopically to visualize the tympanic membrane and to remove wax and any skin desquamation. Using aseptic techniques, the following procedure was applied to both ears:

1 a retro-auricular incision was made, followed by identification of the mastoid bulla;
2 the upper portion of the bulla was opened with a 2mm cutting burr;
3 0.2 ml of propylene glycol 100% was applied using a 25×7 needle on a 1 ml syringe;
4 the skin was closed with Nylon 4–0 sutures;
5 the acoustic canal was inspected with the surgical microscope to identify the tympanic membrane and retro-tympanic fluid.

The right ear was used as the experiment group and the left ear was used as the control group. Immediately following surgery, 0.3 ml of a 25μmol/liter all-trans retinoic acid aqueous solution (0.225g in 20ml solution), equivalent to 4,200 international units (UI/ml), was applied topically with a 1ml syringe in the acoustic canal to cover completely the right tympanic membrane. Saline (NaCl 0.9%) was similarly applied in the left acoustic canal of the same animal.

Topical solutions (all-trans retinoic acid to the right and saline to the left) were applied a second time 48 hours after surgery and subsequently every 48 hours until completing five applications. The animals were contained for 2 minutes during each topical application in both ears to increase the contact between the solution and the tympanic membrane and acoustic canal.

**Figure 1:**
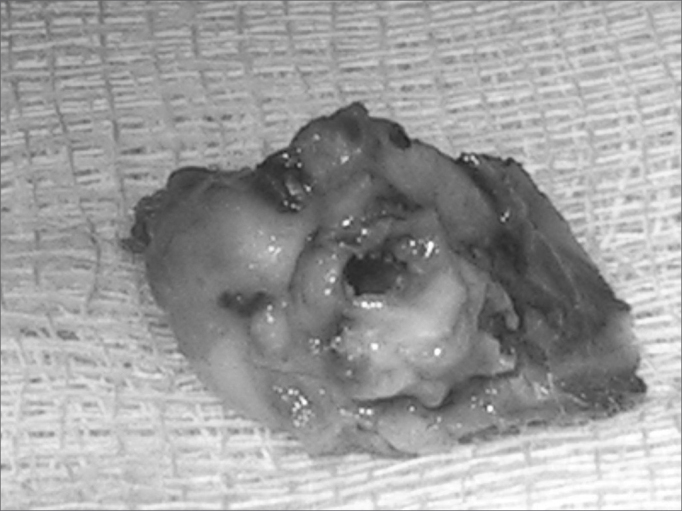
Right temporal bone - note the bulla and the acoustic canal.

The guinea pigs were sacrificed six weeks after the first procedure; this was done by deep anesthesia with ketamine chloridrate (100mg/kg) and xylazine chloridrate (15mg/kg). The tympanic membrane was inspected and the temporal bones were removed (Figure 1). A small opening was made in the bulla if the surgical access route was not identified, for best bone fixation.

**Figure 2:**
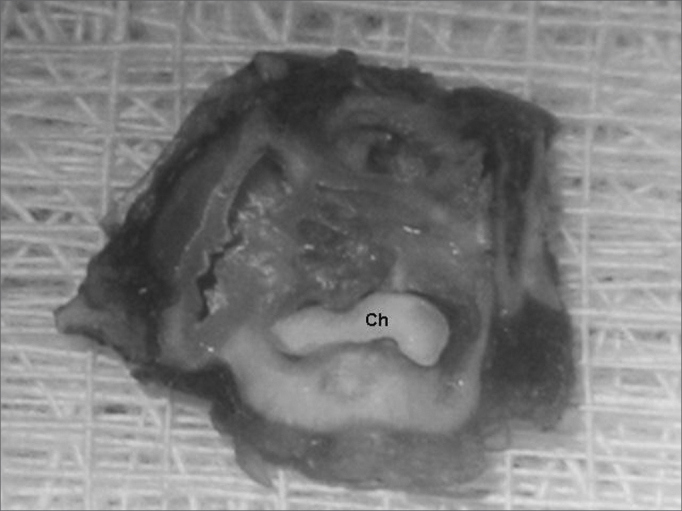
Temporal bone after decalcification and a cross-section - Ch - presence of cholesteatoma (defined macroscopically).

**Figure 3:**
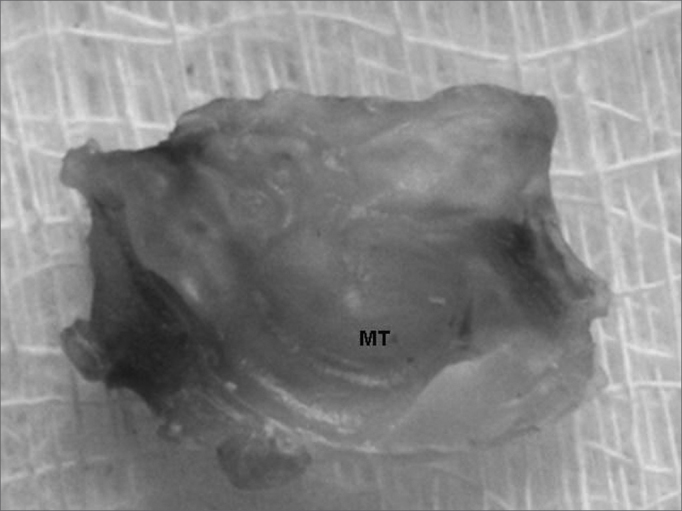
Temporal bone após descalcificação e corte transversal - TM: tympanic membrane. Note the middle ear with no macroscopically observable cholesteatoma.

Each temporal bone was placed in a 10% formaldehyde solution and kept for three weeks for adequate fixation. After fixation, the bones were kept for two weeks in a buffered 12.5% formic acid solution for decalcification. Three millimeter cross-sections of the bones were made parallel to the tympanic membrane to obtain two equally sized specimens for each temporal bone; these were identified as right ear and left ear and section number 1 (lateral, including the bony portion of the acoustic canal and the tympanic membrane) and section number 2 (medial, including the middle ear cavity and the remaining bulla). The sections were analyzed with a surgical microscope at 16 times and 25 times magnification to identify tympanic membrane (TM) and middle ear (ME) changes. Cholesteatomas were defined macroscopically as the presence of a well-organized whitish tumor in the middle ear (Figure 2); possible cholesteatomas were defined as similar but dispersed and suspect areas; no evidence of cholesteatomas was the absence of these changes (Figure 3). Each lateral section, which contained the tympanic membrane and part of the middle ear, was dehydrated and placed in a paraffin block. The next step was to make microtome 3μm cross-sections from which a representative section (involving part of the acoustic canal, tympanic membrane and part of the middle ear) was chosen from each paraffin block. These sections were placed on slides totaling 40 hematoxylin-eosin stained slides from each paraffin block. An expert pathologist analyzed histologically all of the slides; this pathologist did not know which were right or left ears or controls.

**Figure 4:**
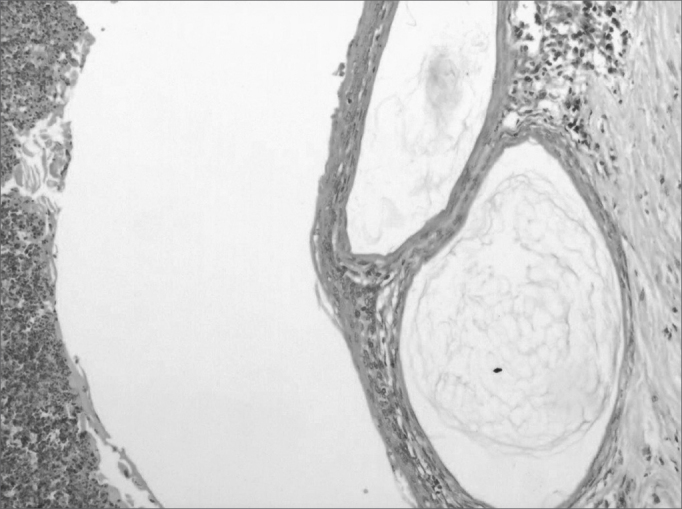
Cholesteatoma cyst in the middle ear (100 X magnification) - note the presence of keratin lamellae within the cholesteatoma matrix.

**Figure 5:**
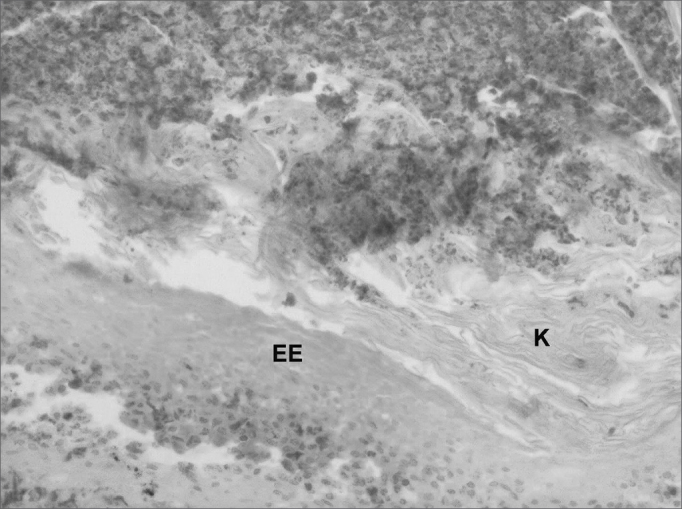
Histological section of a middle ear cholesteatoma (100X magnification) - EE: Epitélio estratificado K: Presence of keratin lamellae.

The histological definition cholesteatoma was the presence of a tumor consisting of non-nucleated keratin scales, and a corneal layer was composed of a stratified squamous epithelium (Figure 4, Figure 5).

Fisher's test was used for analyzing the otoscopic changes seen by using the surgical microscope in relation to each ear. The significance level was 5%.

Fisher's test was also used to analyze the macroscopic findings in specimen temporal bones following its sectioning into two parts. The significance level was 5%.

Fisher's test was used for analyzing the histological findings in right and left temporal bones of 20 guinea pigs in relation to the presence or absence of cholesteatomas. The significance level was 5%.

## RESULTS

Six weeks after applying PG, there remained 20 out of 25 guinea pigs. Two died in the first 24 hours probably due to anesthesia-related problems; the other three died within three weeks probably due to infection. Otoscopy findings are presented on Table 1.

**Table 1:** Otoscopic findings in 20 guinea pigs at the end of the experiment according to each ear.

			Ears			
Findings	Right		Left		Total	
	N	%	N	%	N	%
TM opacity	7	22,59	10	34,48	17	28,33
TM perforation	4	12,90	3	10,34	7	11,67
Acoustic canal secretion	5	16,13	2	6,90	7	11,67
Wax	9	29,04	5	17,25	14	23,33
Acoustic canal polyp	0	0	1	3,44	1	1,67
White retrotympanic tumor (cholesteatoma?)	0	0	4	13,79	4	6,66
Acoustic canal stenosis	1	3,22	0	0	1	1,67
TM hyperemia	4	12,90	2	6,90	6	10,00
TM retraction	1	3,22	2	6,90	3	5,00
Total	31	100,00	29	100,00	60	100,00

**Key:** N = number Test: Fisher's test. NS: Not Significant. TM: tympanic membrane

Statistical analysis (Fisher's test) of the data shown on Table 1 revealed no difference between right and left ears.

Table 2, Table 3 show the findings according to the presence of cholesteatoma, possible cholesteatoma and no evidence of cholesteatoma.

**Table 2:** Temporal bone findings on surgical microscopy in 20 guinea-pigs according to each ear.

			Ears			
Findings	Right		Left		Total	
	N	%	N	%	N	%
No evidence of cholesteatoma	15	75,0	3	15,0	18	45,0
Possible cholesteatoma	2	10,0	2	10,0	4	10,0
Cholesteatoma	3	15,0	15	75,0	18	45,0
Total	20	100,0	20	100,0	40	100,0

**Key:** N = number

**Table 3:** Temporal bone findings on surgical microscopy in 20 guinea-pigs according to each ear after grouping.

	Ears					
Findings	Right		Left		Total	
	N	%	N	%	N	%
No evidence of cholesteatoma	15	75,0	3	15,0	18	45,0
Possible cholesteatoma + Cholesteatoma	5	25,0	17	85,0	22	55,0
Total	20	100,0	20	100,0	40	100,0

**Key:** N = number Test: Fisher's test P= 0.0003 *

There was a statistically significant difference (P=0.0003) between both groups in the distribution of “cholesteatoma” and “possible cholesteatoma” findings compared to the finding “no evidence of cholesteatoma” between right ears (experiment) and left ears (controls). The control group had significantly more suspected findings and/or findings of cholesteatomas compared to the experiment or study group.

**Figure 6:**
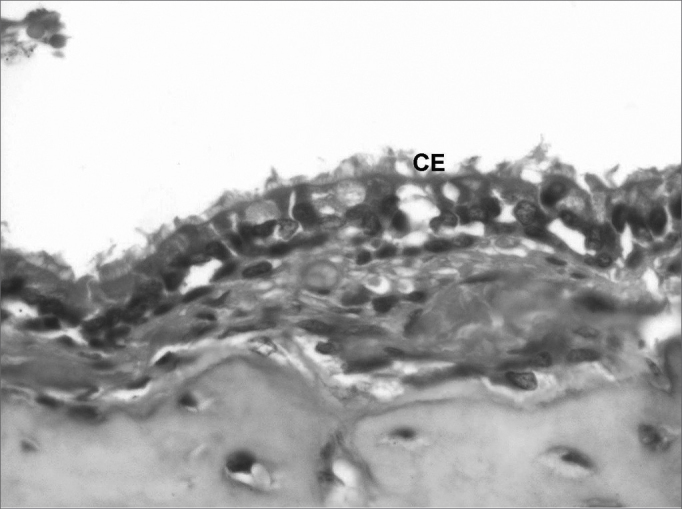
Histological section of the normal middle ear epithelium (400X magnification) - CE: ciliated cylindrical epithelium.

Histological findings of the temporal bones of 20 guinea pigs were divided into two groups, as follows: one group in which histological criteria of cholesteatomas were found, and a second group in which these histological criteria were not found (Figure 6). Table 4 shows the histological results divided into presence and absence of cholesteatomas for each ear.

**Table 4:** Temporal bone histological findings in 20 guinea-pigs according to each ear.

			Ears			
Findings	Right		Left		Total	
	N	%	N	%	N	%
Absence of cholesteatoma	14	70,0	5	25,0	19	47,5
Presence of cholesteatoma	6	30,0	15	75,0	21	52,5
Total	20	100,0	20	100,0	40	100,0

**Key:** N = number Teste: Fisher's test P= 0.0104*

Fisher's test revealed that the right ear had significantly less cholesteatomas compared to the left ear (control).

## DISCUSSION

We chose guinea pigs as an experimental model because of the characteristics of their middle ear, which are similar to those of man. Furthermore, the guinea-pig acoustic canal allows easier inspection and handling such as applying topical substances, which was our case. The bulla is a wide cavity; the tympanic membrane and a non-pneumatic mastoid process form its lateral wall, while its medial wall is formed by most of the middle ear structures including the ossicles, the cochlea, the cochlear and vestibular windows and the semicircular canals.^11^ PG was applied through an access route in the bulla to cause inflammation in the middle ear without handling the tympanic membrane. We wished to analyze the effects of inflammation on the tympanic membrane, especially on epithelial migration. As shown on Table 1, seven ears developed tympanic membrane perforation, while 17 others presented opacity, six developed hyperemia, three developed tympanic membrane retraction and four presented a white retrotympanic tumor (cholesteatoma?). Thus, only three of 40 ears remained normal at the end of the experiment, showing that inflammation caused by PG was significant in most cases.

The most frequent findings were tympanic membrane opacity and the presence of wax. Wax was most evident in the experiment or study ears (right ears), compared to the control ears (left ears). An explanation might be that all-trans retinoic acid at the concentration we used appears to favor wax formation, which was seen immediately after the last application of the substance, differently from the ears that received saline.

PG was recognized as an irritating factor for the middle ear and a possible inducer of cholesteatoma based on observations of optic Cortisporin use, which was a solution that contained PG (10.5%).^3^ Cholesteatoma formation due to PG appears to be dose dependent.^4^^,^^12^ In our study we used PG (100%) to induce cholesteatoma formation in the middle ears of as many animals as possible. We wished to observe and compare whether all-trans retinoic acid, at the chosen concentration and treatment route, would effectively inhibit epithelial migration and, therefore, the development of cholesteatomas.

Is time between PG application and animal sacrifice an important factor in cholesteatoma formation? Some papers have shown variations in histological changes and cholesteatoma formation in groups of animals sacrificed after different intervals. On the third week proliferation of the tympanic membrane epidermal basal layer begins, including papillary growth.^5^^,^^12^ On the sixth week the rate of PG (90%)-induced cholesteatoma reaches 87.5%.^4^ A single application of PG (50%) on the bulla of chinchillas was enough to show evidence of cholesteatoma formation on the third week.^3^ The time, therefore, between applying the mucosal irritant and sacrificing the animal is important for cholesteatoma formation; from the third week onwards epithelial migration and a typical cholesteatoma have been formed, in most papers. The moment for analyzing the specimens histologically varied according to different authors and cholesteatoma-inducing techniques; we chose six weeks, similar to some other authors, as we believe that this period is sufficient for a cholesteatoma to form. A longer period before histological analysis increases the morbidity and mortality of the specimens due to complications of the cholesteatoma itself or associated infections.

The pathogenesis of acquired cholesteatoma is based mainly on metaplasia or migration. This, in turn, may be subdivided into three categories, as follows: migration through a tympanic membrane perforation; invagination, characterized by progressive tympanic membrane invagination; and papillary growth, in which the epidermal layer of the tympanic membrane grows into the middle ear.^13^ Theses description have also been published by other authors.^3^^,^^14^

The tympanic membrane alterations we found in 40 ears (control and experiment groups) included visible retro-tympanic cholesteatomas in four ears, of which two presented retraction, and tympanic membrane perforation in seven ears. We were able, therefore, to observe cholesteatoma formation in retracted, intact and perforated tympanic membranes; perforation might be secondary to necrosis, itself resulting from inflammation caused by the middle ear mucosa-irritating agent.

Table 1 summarizes the otoscopic findings at the end of the experiment, demonstrating that there were more visible retro-tympanic cholesteatomas in the control group, although this difference was not statistically significant. Otoscopy of the control group revealed tympanic perforation in three ears, and tympanic membrane retraction was observed in two ears (Table 1). Macroscopy of temporal bone section revealed cholesteatomas in 17 ears. Most of the cholesteatomas formed regardless of any tympanic membrane perforation or retraction; if retraction was present in the control ears, however, a cholesteatoma was more easily seen otoscopically. The fact that most of the ears that developed cholesteatomas in the control group did not present tympanic membrane perforation strengthens the epithelial migration theory. We believe that tympanic membrane epidermal layer basal cell proliferation is an initiating factor for cholesteatoma formation.

Various papers reported unsuccessful attempts to inhibit cholesteatoma formation, the so-called “prophylactic therapy” of cholesteatoma.^8^^,^^9^ There are a number of possible explanations for these findings, such as the use of a vitamin A analogue. One possibility is that isotretinoin treatment was done prior to the use of an irritating agent; findings might have been different if both agents had been used simultaneously. A second possibility is that the drug was used systemically; if it had been used topically, it might have more effectively inhibited epithelial migration. Another possibility is that isotretinoin is not effective against migration at the dose that was used, or even regardless of the dose.

Hyaluronic acid^7^ has been used topically to inhibit PG-induced cholesteatoma formation in chinchillas; in this study, cholesteatomas were found in 70% of the ears treated with hyaluronic acid and in 71% of the untreated ears. Extracellular calcium appears to be an important factor both for cell migration and adhesion in cholesteatomas. If calcium is removed from cholesteatoma growth and migration stimulating culture media, there is a tenfold reduction in the degree of cholesteatoma migration.^15^ Calcium is essential for cell to cell adhesion, for instance, between keratinocytes and substrate cells. These authors studied the in vitro inhibitory effects of all-trans retinoic acid on cholesteatoma migration in human cholesteatoma samples cultured in cell-growth simulating media. The maximum effect was six times more inhibition of migration at a higher concentration of all-trans retinoic acid (5 μmol/liter). It appears that the main effects of all-trans retinoic acid on cholesteatoma migration are decreased cell adhesiveness and cytoskeleton alterations, including altered calcium concentrations.^10^ All-trans retinoic acid is widely used in dermatology; its effects include reducing epithelial cell adhesiveness and treating skin keratinization disorders, including repairing skin after exposure to phototherapy and other external agents. It is used for the treatment of acne vulgaris and psoriasis, among other conditions.

An attempt to inhibit epithelial migration with all-trans retinoic acid seemed pertinent based on the abovementioned findings and the lack of animal studies with an adequate methodology to attempt in vivo migration. The authors of another study that used topical vitamin A induced cholesteatoma formation by ligating the acoustic canal; they found no statistically significant difference between the vitamin A and Cortisporin (without PG) treated groups. A different was found when comparing the treated groups with controls; in this study, cholesteatoma formation in the treated group was 60%–65%.16 The method did not clarify how many substance applications were made, or whether the treatment lasted throughout the nine months that the experiment was conducted. Furthermore, it is difficult to apply a topical substance in a surgically closed cavity, since wax and debris accumulate and fibrosis sets in, all of which may have altered the effect of the substances. A further point is that the surgical procedure to ligate the acoustic canal may alter the effect of those tested substances on the various epidermal layers of the acoustic canal by altering its anatomy and physiology, thereby affecting the results. We believe, therefore, that an experimental method that does not alter the anatomy of the external ear, and specifically the acoustic canal, would be more appropriate and would not interfere on the effect of cholesteatoma formation inhibiting substances. We found moderate quantities of wax in some ears with our method, particularly in the experimental or study ears. The three ears in this group that presented a cholesteatoma developed wax, secretion and tympanic membrane perforation. This may have influenced cholesteatoma formation by decreasing external ear ventilation and reducing the contact time between all-trans retinoic acid and the tympanic membrane epithelial layer, possibly altering substance absorption.

Table 2, Table 3 present the findings “possible cholesteatoma”, “cholesteatoma” and “no evidence of cholesteatoma”, revealing that the control group had a significantly higher number in the cholesteatoma and possible cholesteatoma categories. Confronting these findings with the temporal bone histological analysis revealed that the macroscopic definition of cholesteatoma was well applied, as all of the temporal bones (n=18) that fit into this macroscopic definition also fulfilled the histological criteria for cholesteatoma (presence of a stratified squamous epithelium covered by keratin scales). Some of the bones that were macroscopically classified under the category “no evidence of cholesteatoma” had histological features of cholesteatomas. The macroscopic category “no evidence of cholesteatoma” was seen in 18 ears, of which three were identified histologically as having middle ear cholesteatoma. Based on the definitions we used, if a cholesteatoma was defined macroscopically, histology confirmed its presence in 100% of cases, whereas if the macroscopic definition was “no evidence of cholesteatoma”, cholesteatomas were found in 16.7% of cases; thus, it is important to compare macroscopic and histological findings. The remaining 83.3% of cases with no macroscopic evidence of cholesteatomas in fact did not show any histological finding on the slides. There were no histological findings of cholesteatomas in the group of ears macroscopically classified as “possible cholesteatoma” (n=4). The possibility of cholesteatomas was not confirmed; the finding in some slides of small whitish areas dispersed in the temporal bone (as defined macroscopically) may be inflammatory alteration of the middle ear.

Table 4 presents histological findings showing that cholesteatomas were present in 30% of the ears in the group treated with all-trans retinoic acid (6/20) and in 75% of the control ears (15/20). Statistical analysis (Fisher's test) found that the presence of cholesteatomas in the group of ears treated with all-trans retinoic acid was significantly lower compared to the control ears (p=0.0104); all-trans retinoic acid, therefore, effectively inhibited cholesteatoma formation in our model.

Our findings differed from those of other authors16 that used vitamin A topically to inhibit cholesteatoma formation. We believe that this difference may be explained by the concentration we used, the method (as discussed above), and possibly by different pharmacological effects between retinoids.

Cholesteatoma inhibition by all-trans retinoic acid in out study was concordant with findings of in vitro inhibition by this drug.^10^ This particular paper fundamentally helped us decide in favor of all-trans retinoic acid as a cholesteatoma formation inhibitor; we believe that the application method and the drug concentration were responsible for our results. Wax and/or secretion in some of the all-trans retinoic acid treated ears may have altered its local effect, which would explain the presence of cholesteatomas in some of the treated ears.

These comments and our results suggest that further studies are needed to apply these findings and others that might arise, such as the effect of all-trans retinoic acid on the keratonicyte cell structure, to investigate the use of this drugs on humans.

## CONCLUSION

Our results allow us to conclude that topical use of all-trans retinoic acid applied to the acoustic canal and the area adjacent to the tympanic membrane is an effective inhibitor of propylene glycol-induced cholesteatoma formation in the middle ear of guinea pigs.
